# Hepatic steatosis: Qualitative and quantitative sonographic assessment in comparison to histology

**DOI:** 10.1002/ajum.12381

**Published:** 2024-04-25

**Authors:** Zhi Xin Tan, Bryan Mehta, Kieran Kusel, James Seow, Marilyn Zelesco, Steven Abbott, Rebecca Simons, Glenn Boardman, Christopher J. Welman, Oyekoya T. Ayonrinde

**Affiliations:** ^1^ Gastroenterology and Hepatology Fiona Stanley Hospital Murdoch Western Australia Australia; ^2^ Department of Medical Imaging Fiona Stanley Hospital Murdoch Western Australia Australia; ^3^ Department of Radiology Royal Perth Hospital Perth Western Australia Australia; ^4^ SMHS Research Support and Development Unit Murdoch Western Australia Australia; ^5^ Medical School The University of Western Australia Crawley Western Australia Australia; ^6^ Faculty of Health Sciences Curtin University Perth Western Australia Australia

**Keywords:** attenuation imaging, hepatic steatosis, histopathology steatosis grading, ultrasound parameters

## Abstract

**Introduction:**

Globally, B‐mode ultrasound is the most common modality used for the diagnosis of hepatic steatosis. We aimed to assess the correlation between qualitative liver ultrasound parameters, attenuation imaging (ATI) and histopathology‐diagnosed steatosis grade obtained from liver biopsy. Our secondary aim was to examine the interobserver variability of qualitative ultrasound features.

**Methods:**

A retrospective cohort study was performed which included adult patients (age ≥ 18 years) who had same‐day liver ultrasound, ATI and liver biopsy for grading hepatic steatosis severity between 2018 and 2022. The qualitative US features for hepatic steatosis were independently scored by three radiologists and interobserver variability was examined. Histologic steatosis grade, ATI and qualitative ultrasound parameters were compared.

**Results:**

Ninety patients were included; 67% female with a median age of 54 (IQR 39–65) years. The radiologist's overall impression had the highest correlation (very strongly correlated) with histologic steatosis grade (r = 0.82, P < 0.001). ATI coefficient and all qualitative ultrasound B‐mode features except for liver echotexture and focal fat sparing were strongly correlated with histologic steatosis grade (r ≥ 0.70, P < 0.001). Most qualitative ultrasound features had good agreement between observers (Kappa statistic 0.61–1.0, P < 0.001), (Kendall coefficient 0.92, P < 0.001).

**Conclusion:**

The examined qualitative ultrasound parameters and ATI had good‐excellent performance for diagnosing clinically significant hepatic steatosis; however, the radiologist's overall impression had the best correlation with histologic steatosis grade. Our findings suggest an ongoing role for qualitative liver ultrasound assessment of hepatic steatosis despite the emergence of newer quantitative measures.

## 1 Introduction

Steatotic liver disease (SLD), formerly known as fatty liver, is the most common chronic liver disease worldwide.^1^ Recent consensus guidelines recommend metabolic‐dysfunction associated steatotic liver disease (MASLD) as the preferred nomenclature to provide a non‐stigmatising descriptor, replacing non‐alcoholic fatty liver disease (NAFLD).^2^ It is defined by hepatic steatosis plus at least one cardiometabolic risk factor. In the last decade, NAFLD was the most common indication for liver transplantation in younger patients in Australia and New Zealand.^3^, ^4^ Given the potential for severe complications, several diagnostic approaches aim to identify NAFLD early to alter the trajectory away from progression to cirrhosis and liver‐related complications.^5^, ^6^, ^7^

While the World Global Gastroenterology Organisation discourages general population screening for NAFLD, diagnostic tests are recommended for individuals with risk factors such as diabetes and obesity.^6^, ^7^ Liver biopsy for histological analysis has traditionally been the gold standard for diagnosis and grading of hepatic steatosis but due to the associated risks, higher cost and lower availability, it is generally reserved for high‐risk cases or as an inclusion criterion for clinical trials (Table 1).^8^ Furthermore, liver biopsy may be inaccurate due to sampling variability.^8^, ^9^, ^10^ While magnetic resonance imaging (MRI) proton density fat fraction (MRI‐PDFF) is the most accurate non‐invasive tool for hepatic steatosis assessment, its widespread use is hindered by limited availability and high cost.^6^, ^7^, ^11^ Hence, the recommended diagnostic workup for suspected MASLD by the American Association for the Study of Liver Disease (AASLD) 2023 guidance recommends controlled attenuation parameter (CAP) as the primary tool for measuring hepatic steatosis.^5^ Additionally, B‐mode ultrasonography or ultrasound attenuation methods are suggested for hepatic steatosis work‐up due to their greater accessibility, safety and of costeffectiveness.^5^, ^6^, ^7^, ^12^

**Table 1:** Brunt steatosis grade^1^.

Brunt steatosis grade (Histopathology analysis)	Percent of fat present in hepatocytes/Steatosis percentage in examined sample
S0	<5%
S1	5–33%
S2	>33%–66%
S3	≥66%

Ultrasound assessment for hepatic steatosis can be broadly categorised into qualitative ultrasound B‐mode imaging, semiquantitative methods such as the Hamaguchi score and Hepatorenal‐index (HRI) and lastly quantitative tools including CAP, ultrasound attenuation methods such as attenuation imaging (ATI), attenuation measurement function (ATT), ultrasound‐guided attenuation parameter (UGAP), backscatter coefficient (BSC) and speed of sound.^13^, ^14^

### 1.1 Qualitative ultrasound

A meta‐analysis demonstrated that B‐mode ultrasound had a pooled sensitivity and specificity of 84.8% and 93.6% with an AUROC of 0.93 in detecting hepatic steatosis of any cause. The sensitivity and specificity increased to 100% and 90%, respectively, when there was > 20% hepatic steatosis.^13^, ^15^ A recent study further highlighted the diagnostic accuracy of B‐mode US, revealing an AUROC of 0.861 and 0.923 for S2 and S3, respectively (Table 2).^16^

**Table 2:** Correlation of US parameters with hepatic steatosis grades according to histopathology steatosis grade and controlled attenuation parameter steatosis grade.

Qualitative ultrasound parameters	Reference standard	S1	S2	S3
US B‐mode imaging	Biopsy^16^	Sensitivity: 75.6% Specificity: 76% AUROC: 0.798	Sensitivity: 90.9% Specificity: 64.6% AUROC: 0.861	Sensitivity: 89.5% Specificity: 89.9% AUROC: 0.923
Hamaguchi score	Biopsy^19^	Sensitivity: 91.2%–92.6% Specificity: 100% AUROC: 0.980		
	CAP^21^	Sensitivity: 86.9% Specificity: 100%	Sensitivity: 79.8% Specificity: 97.5%	Sensitivity: 72.9% Specificity: 97.2%
HRI	Biopsy^20^	Sensitivity: 100% Specificity: 91% AUROC: 0.992	Sensitivity: 90% Specificity: 90% AUROC: 0.960	Sensitivity: 90% Specificity: 93% AUROC: 0.957
	CAP^21^	Sensitivity: 91.6% Specificity: 86.2%	Sensitivity: 94% Specificity: 80.2%	Sensitivity: 57.6% Specificity: 90.6%

CAP, controlled attenuation parameter; HRI, hepatorenal‐index.

B‐mode ultrasound allows subjective grading of hepatic steatosis as absent, mild, moderate or severe (Table S1). The widely used grading system involves comparing the liver's echogenicity to the renal cortex, evaluating the visualisation of hepatic vessel walls and the diaphragm, and assessing posterior beam attenuation.^13^, ^15^, ^17^, ^18^ Previous studies reported reduced diagnostic efficacy in cases of mild hepatic steatosis, high body mass index (BMI) and hepatitis C infection. However, advancements in ultrasound technology have mitigated the impact of BMI on the accuracy of hepatic steatosis diagnosis.^16^

### 1.2 Semi‐quantitative ultrasound

The Hamaguchi method utilises subjective parameters on B‐mode ultrasound, assigning a score from 0 to 6 based on four main parameters (Table 2). It has an AUROC of 0.98 for a score > 2 for steatosis diagnosis with high sensitivity and specificity of 91.7% and 100%, respectively (Table 2). Hamaguchi score proves effective in reducing variability, with an interobserver agreement of 0.95.^19^

HRI measures the B‐mode liver/right kidney cortex echogenicity ratio in terms of image pixel brightness, unaffected by the degree of fibrosis or steatohepatitis, showing a good interobserver agreement of 0.956 However, its reliance on normal renal cortical echogenicity limits its use in conditions with increased renal echogenicity. For all steatosis grades, the AUROC of HRI is >0.90 (Table 2).^16^, ^19^, ^20^, ^21^

### 1.3 Quantitative ultrasound

Controlled attenuation parameter and ATI are the more widely used quantitative ultrasound techniques to assess the severity of hepatic steatosis.^5^, ^13^, ^21^, ^22^, ^23^, ^24^ FibroScan® CAP measures signal amplitude loss (i.e. attenuation) of the ultrasound wave as it passes through liver parenchyma. Published CAP cut‐off values based on steatosis grade are listed in Table S4. It demonstrates good correlation with other non‐invasive indices including Hamaguchi Score and HRI, and its AUROC for detecting steatosis ≥ S1 is >0.80.^21^

Similar to CAP, ATI assesses ultrasound beam attenuation using B‐mode ultrasound images to select the region of interest. ATT and UGAP also leverage US guidance to assess the attenuation coefficient of the region of interest. However, CAP's drawback lies in its lack of US guidance when selecting the measurement area.^14^ Suggested cut‐off values for ATI are listed in Table S5. With an AUROC of >0.90 for all grades of hepatic steatosis with high correlation with histological grading and MRS findings, ATI accurately discriminates mild from moderate steatosis.^22^, ^23^, ^24^ Comparative studies with CAP showed ATI's higher AUROC and better correlation with MRI‐PDFF.^25^ Additionally, ATI demonstrates higher correlation with liver biopsy but no significant difference in AUROC compared to CAP.^26^ Evaluated against MRI PDFF, ATI and HRI both demonstrated reasonable accuracy in grading hepatic steatosis; however, HRI exhibited a superior AUROC compared to ATI.^27^

Recent comparative studies among quantitative parameters including CAP, ATI, tissue attenuation imaging (TAI) and tissue scatter‐distribution imaging (TSI) show AUROC of >0.8 in detecting hepatic steatosis ≥5%, with CAP, ATI and TSI displaying superior AUROC to TAI for detecting hepatic steatosis ≥10%. Combining parameters (TSI + TAI) yields the highest AUROC suggesting the potential utility of employing multiple quantitative parameters to improve hepatic steatosis evaluation.^28^

There is currently no study comparing the relative accuracy of ATI and qualitative greyscale features in grading hepatic steatosis against liver biopsy. The aim of this study was therefore to compare the correlation between ATI and qualitative B‐mode features, using liver biopsy as the reference standard in an adult population.

### 1.4 Aims

Primary aim: To assess the correlation of a quantitative US parameter (ATI) and qualitative B‐mode US features for hepatic steatosis assessment using liver biopsy as the reference standard.

Secondary aim: To investigate the interobserver variability of qualitative ultrasound B‐mode features for hepatic steatosis assessment.

## 2 Method

### 2.1 Participants

Single centre retrospective cohort study with adult patients (age ≥ 18 years) referred to the Medical Imaging Department for liver biopsy from 2018 to 2022 (census date) screened for eligibility. Only patients with a hepatic steatosis grade on histopathology and with US imaging performed in conjunction with US‐guided biopsy were included.

This study was approved by the Western Australia South Metropolitan Health Service low‐risk ethics committee Governance, Evidence, Knowledge, Outcome (GEKO) as this was a low‐risk retrospective study and data were analysed in a de‐identified manner. Reference number: GEKO 46317.

### 2.2 Data collection and extraction

Patient data were sourced from hospital medical records while B‐mode ultrasound images and ATI were reviewed on the hospital imaging PACS/RIS (version 6.7.0.6011; Agfa, Mortsel, Belgium). Liver biopsy histology results were obtained from the laboratory database.

Patients' data collected include demographics (age, sex) and body mass index (BMI). Relevant medical history included alcohol use and the presence or absence of type II diabetes mellitus, hypertension, ischemic heart disease, dyslipidaemia and gallbladder pathology. Ischemic heart disease is based on a documented history of existing coronary artery disease, diagnosed by coronary artery imaging or a previous acute coronary event. Dyslipidaemia was defined as raised total or LDL cholesterol, hypertriglyceridemia or low HDL cholesterol. Gallbladder pathology is based on the presence of gallstones and/or gallbladder polyps seen on imaging, or previous documented choledocholithiasis or cholecystectomy.

Liver ultrasound imaging was obtained on the same day as liver biopsy. Ultrasound parameters obtained were skin‐to‐liver capsule distance (SCD), SWE, ATI, HRI and shear wave dispersion (SWD). Liver biopsy results including fibrosis and steatosis grade were recorded.

Subjective ultrasound parameters were recorded by retrospective analysis of the ultrasound images. Large hepatic vein blurring (LHVB), main and right portal vein blurring, anterior division and posterior division right portal vein blurring, liver–kidney contrast, posterior beam attenuation, diaphragm definition, focal fat sparing, liver echotexture and overall impression were recorded for each patient using set criteria (Table S6).

### 2.3 Acquisition and interpretation of images

Three sonographers (M.Z. – 36 years, S.A. – 13 years and R.S.M – 2 years of experience) were involved in compiling patients' images of interest. The included ultrasound study for each patient has between 20 to 60 images however only 4 to 10 are selected to answer the research question.

Three radiologists (C.J.W. – 24 years, J.S – 17 years and K.K. – 4 years of experience) that were blinded to clinical data reviewed ultrasound images in PACS and graded qualitative images according to set criteria (Tables S2 and S6). Image analysis was performed for the purposes of this study and was independent of the existing ultrasound report.

Images were obtained using a Canon Aplio i‐series (i700, i800) US system (Canon Medical Systems, Otawara, Japan) with an 8C1 3.5 MHz transducer.

### 2.4 Histopathological analysis

Standard 16‐18G Temno (Merit, USA) or Biopince (Argon medical Devices, Plano, TX, USA) core samples were obtained from the right liver lobe (segment 6 or 7) at the same appointment, immediately following imaging acquisition. At least 2 core samples were obtained, and adequate samples required were length >20 mm, diameter >1.4 mm and >11 portal tracts visualised. Samples were graded using Brunt and METAVIR scores.

Three pathologists were involved in the grading of the liver biopsies (pathologist A – 2 years, pathologist B – 13 years and Pathologist 3–18 years of experience as anatomical pathologist).

### 2.5 Statistical analysis

Patient baseline characteristics and ultrasound parameters were expressed with descriptive statistics as either number with percentage (%), mean with standard deviation (SD) or median with interquartile range (IQR).

Spearman rank coefficient was used to investigate the correlation between the ultrasound parameters and the histology steatosis grade, expressed as r. Interobserver agreement of subjective ultrasound parameters was assessed using Cohen Kappa coefficient and Kendall's coefficient for 2 and 3 observers, respectively. P values <0.001 were considered significant in all analyses.

Software used for analysis included R by R Core Team 2021 and Hmisc: Harrell Miscellaneous. R package version 5.0‐1.

## 3 Results

**Figure 1:**
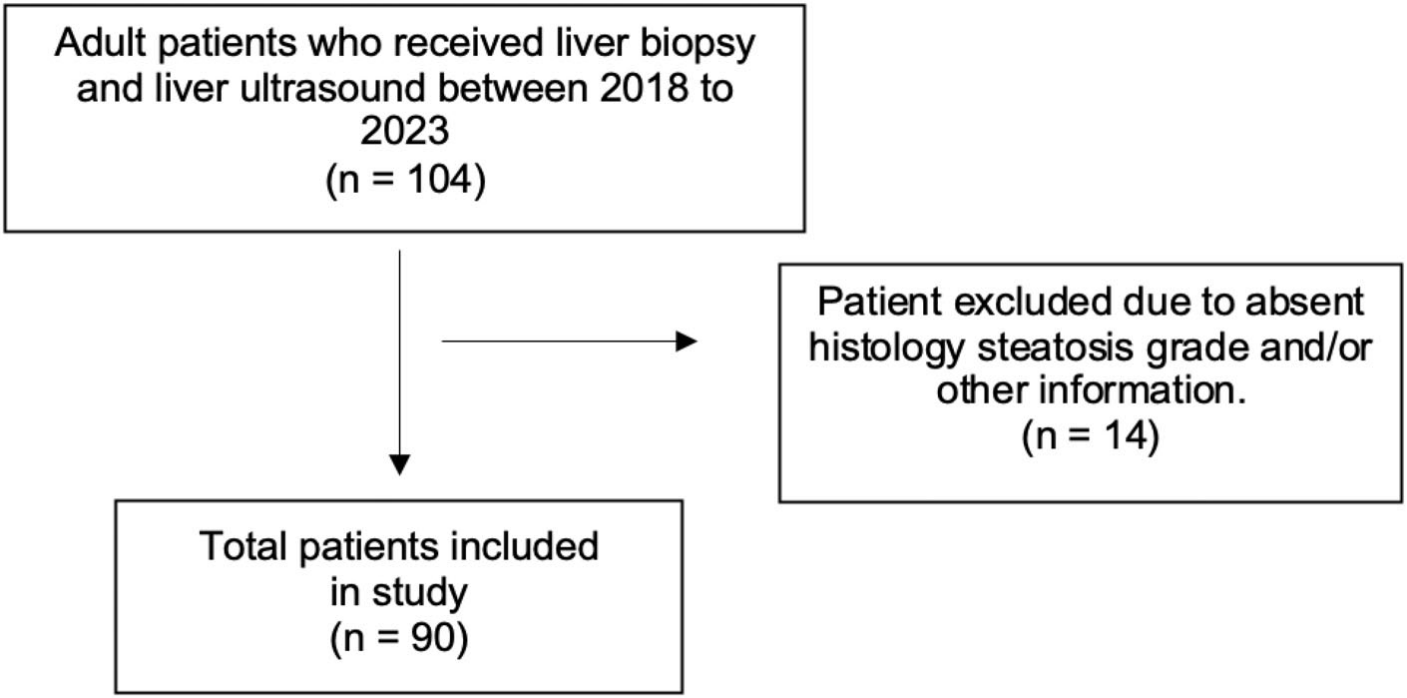
Patient inclusion flowchart.

Of the 90 patients included (Figure 1), 67% were female with a median age of 54 years (IQR 41–64). Mean patient BMI was 30.8 kg/m^2^. Table 3 shows patient demographics and relevant medical history. Majority of the patients have a histologic fibrosis score of Metavir F1 (49%) and steatosis score of S0 (31%), respectively. The most common indications for liver ultrasound and liver biopsy are summarised in Table 3.

**Table 3:** Data are presented as mean (±SD), number (%) or median (IQR). Patient baseline characteristics and ultrasound parameters.

Characteristics	Distribution
Female, n (%)	60 (67%)
Age, years, median (IQR)	54 (39–65)
BMI, kg/m^2^, mean (SD)	30.8 (±9.09)
Past medical history, n (%)	
Excessive alcohol consumption^a^	5 (5.6%)
Type 2 diabetes mellitus	29 (32.2%)
Dyslipidaemia	30 (33.3%)
Ischemic heart disease	6 (6.7%)
Gallbladder pathology^b^	8 (13.3%)
Indication for liver biopsy, n (%)	
Nonalcoholic steatohepatitis	40 (45%)
Autoimmune hepatitis	12 (13%)
Drug‐induced liver injury	8 (9%)
Primary biliary cholangitis/primary sclerosing cholangitis	12 (13%)
Viral hepatitis	5 (6%)
Other^c^	13 (14%)
Non‐steatosis US assessment, median (IQR)	
TE, kPa	9.3 (IQR: 6.5–21)
SWE, kPa	7.5 (IQR: 5.7–11)
SCD, mm	23 (±8.9)
SWD, [m/s]/kHz	12.5 (IQR: 9.98–15.3)
Steatosis US assessment, median (IQR)	
ATI, dB/cm/MHz	0.725 (IQR: 0.62–0.86)
CAP, dB/m	316 (IQR 271–361)
METAVIR score, n (%)	
F0	15 (17%)
F1	44 (49%)
F2	7 (8%)
F3	13 (14%)
F4	11 (12%)
Brunt steatosis score, n (%)	
S0	37 (41%)
S1	17 (19%)
S2	20 (22%)
S3	16 (18%)

AIH, autoimmune hepatitis; ATI, attenuation imaging; CAP, controlled attenuation parameter; DILI, drug‐induced liver injury; HRI, hepatorenal‐index; IQR, interquartile range; NASH, nonalcoholic steatohepatitis; SCD, skin‐to‐liver capsule distance; SWE, shear wave elastography; SWD, shear wave dispersion; TE, transient elastography.

^a^ Excessive alcohol consumption is defined as more than 21 standard drinks and 14 standard drinks per week for men and women, respectively.

^b^ Presence of cholelithiasis or previous cholecystectomy.

^c^ Other indications include liver sarcoidosis, graft‐versus‐host disease and alcohol liver disease.

**Figure 2:**
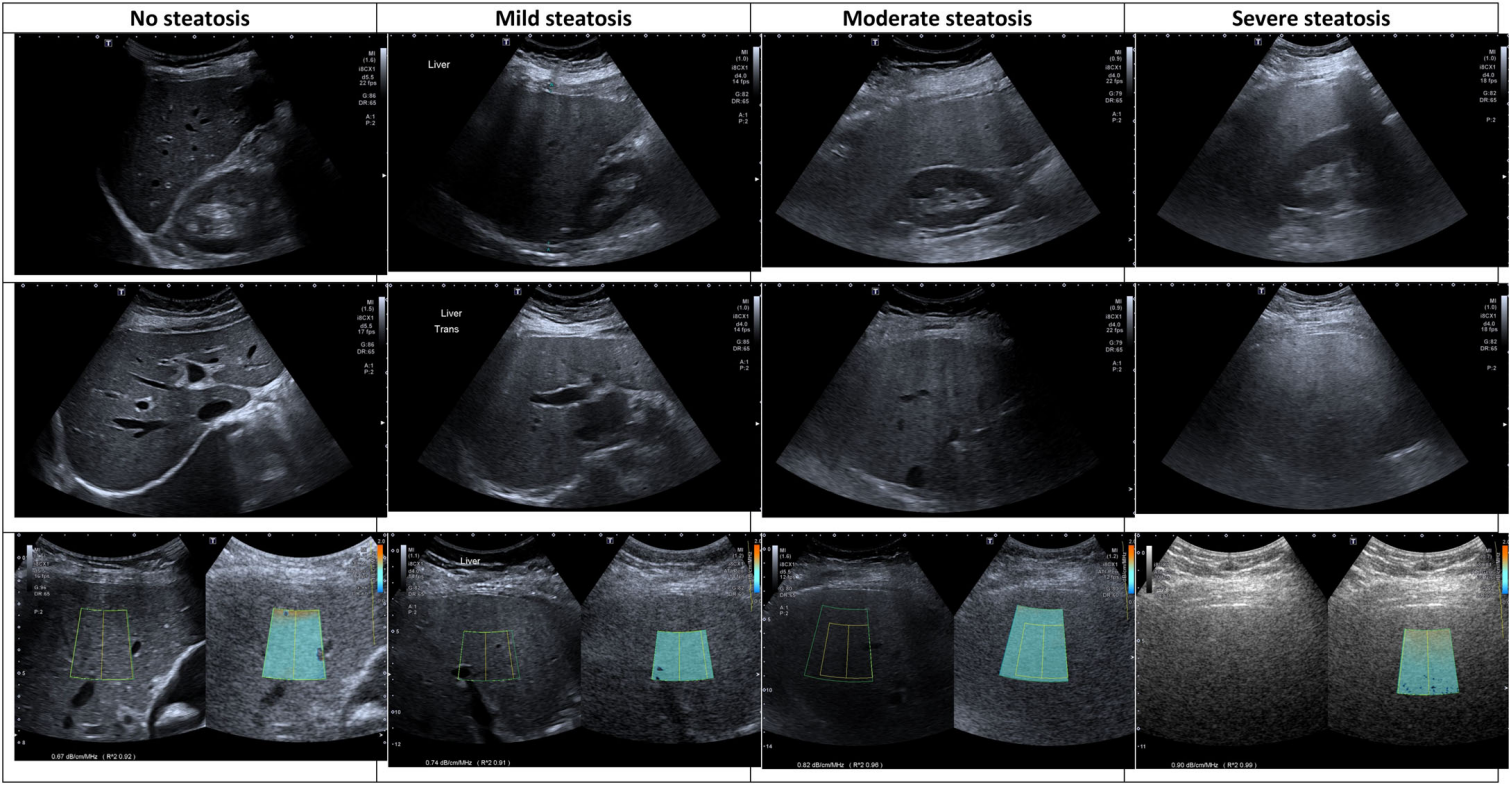
Selected ultrasound images from study patients with no steatosis, mild steatosis, moderate steatosis and severe steatosis (left to right) based on the Radiologist's overall impression. Images in the top row demonstrate liver–kidney contrast, and images in the middle row highlight vessel wall blurring, posterior beam attenuation and diaphragmatic definition. Images in the bottom row demonstrate a single attenuation imaging measurement for each patient, in these examples: 0.67, 0.74, 0.82 and 0.90 dB/cm/MHz respectively. Note that the *R* ^2^ values are ≥0.90, indicating quality measurement.

For the primary aim, the radiologist's overall impression had the highest Spearman correlation coefficient of 0.82 (CI: 0.74–0.88, P < 0.001) with histology steatosis grade (Table 4). This indicates a strong monotonic relationship between the two variables as the closer the number is to 1, the stronger the relationship between the two variables. Examples for radiologist's overall impression grading are illustrated in Figure 2. This was followed closely by liver–kidney contrast, r = 0.80, (CI: 0.69–0.87, P < 0.001), Hamaguchi score and bright liver–kidney contrast, both with r = 0.79, P < 0.001. In contrast, the lowest correlation with histology analysis was liver echotexture with r = −1.09, P = 0.74. We note that focal fat sparing had a weak correlation of 0.23 with a statistically insignificant P value of 0.514. The other B‐mode US features displayed a strong correlation with histology grade with r > 0.70, P < 0.001.

**Table 4:** Spearman correlation between Brunt steatosis grade and US parameters.

Ultrasound parameter	Liver histology steatosis grade		
	Spearman correlation coefficient r	95% confidence interval	P value
Radiologist overall impression	0.82	0.74,0.88	<0.001
ATI	0.70	0.58,0.80	<0.001
Hamaguchi score	0.79	0.69,0.86	<0.001
Bright liver and hepatorenal contrast	0.79	0.69,0.86	<0.001
Deep attenuation	0.78	0.67,0.85	<0.001
Vessel blurring	0.74	0.62,0.83	<0.001
LHVB	0.75	0.63,0.88	<0.001
Liver–kidney contrast	0.80	0.69,0.87	<0.001
Posterior beam attenuation	0.70	0.56,0.79	<0.001
Diaphragm definition	0.73	0.60,0.82	<0.001
Main and right PVB	0.76	0.65,0.84	<0.001
Focal fat sparing	0.23	0.09,0.37	0.051
Liver echotexture	−1.09	−0.27,0.07	0.320

ATI, attenuation coefficient; LHVB, large hepatic vein blurring; Main and right PVB, main and right portal vein blurring.

Bright liver and hepatorenal contrast and liver–kidney contrast had the best correlation with the radiologist's overall impression (r = 0.93, P < 0.001), followed by Hamaguchi score, which had a very strong correlation (r = 0.90, P < 0.001) (Table 5). ATI also had a strong correlation with overall impression with (r = 0.75, P < 0.001). The other examined variables also displayed good correlation with r ≥ 0.75. Liver echotexture was poorly correlated with the radiologist's overall impression.

**Table 5:** Spearman correlation between radiologist's overall impression, ATI and other ultrasound parameters.

Radiologist's overall impression			
Ultrasound parameter	Spearman correlation coefficient r	95% confidence interval	P value
ATI	0.75	0.64,0.83	<0.001
Hamaguchi score	0.91	0.87,0.94	<0.001
Bright liver and hepatorenal contrast	0.93	0.89,0.96	<0.001
Deep attenuation	0.84	0.76,0.89	<0.001
Vessel blurring	0.80	0.70,0.86	<0.001
LHVB	0.84	0.74,0.90	<0.001
Liver–kidney contrast	0.93	0.89,0.95	<0.001
Posterior beam attenuation	0.75	0.64,0.83	<0.001
Diaphragm definition	0.77	0.66,0.85	<0.001
Main and right PVB	0.84	0.76,0.90	<0.001
Liver echotexture	−0.04	−0.25,0.18	0.740

ATI, attenuation coefficient; LHVB, large hepatic vein blurring; Main and right PVB, main and right portal vein blurring.

Owing to the retrospective nature of the study, some patients did not have images for some subjective ultrasound features that resulted in varying numbers in the graded cases (Table 6). Among the B‐mode features, the kappa value was > 0.80 for deep attenuation, vessel blurring, LHVB, main and right portal vein blurring and posterior beam attenuation, indicating high interobserver agreement. Bright liver & hepatorenal contrast, liver–kidney contrast and diaphragm definition had substantial agreement with kappa values of 0.60–0.80. Liver echotexture had moderate agreement between observers with a kappa value of 0.582. Hamaguchi score had a low interobserver agreement of 18.5%, possibly because it has a wide score range (0–7) resulting in a greater chance of interobserver disagreement. About 41.5% of overall impression ratings were identical among the 3 observers with near perfect interobserver agreement with Kendall's concordance coefficient of 0.90, P < 0.001.

**Table 6:** Interobserver rating of subjective ultrasound parameters using Kappa Statistics.

Ultrasound parameter	Number of cases	% Agreement between 2 observers	Kappa value	P value
Bright liver & hepatorenal contrast	80	72.5	0.729	<0.001
Deep attenuation	82	90.2	0.844	<0.001
Vessel blurring	82	92.7	0.851	<0.001
Hamaguchi score	81	18.5	−0.090	0.188
LHVB	66	93.9	0.869	<0.001
Main and right portal vein blurring	77	89.6	0.814	<0.001
Liver–kidney contrast	79	70.9	0.698	<0.001
Posterior beam attenuation	82	89.0	0.833	<0.001
Diaphragm definition	79	87.2	0.777	<0.001
Focal fat sparing	67	91.0	−0.041	0.718
Liver echotexture	81	93.8	0.582	<0.001

LHVB, large hepatic vein blurring.

## 4 Discussion

Steatotic liver disease has been recognised as a potential cause of progressive liver disease for centuries, now emerging as the primary cause for liver mortality and morbidity.^1^, ^29^, ^30^ Beyond liver implications, individuals with MASLD have an increased risk of developing cardiovascular disease and chronic kidney disease.^3^, ^11^, ^31^ Various studies dating back to the early 1900s and more detailed studies in recent years consistently show the correlation between severity of hepatic steatosis and the heightened occurrence of complications.^1^, ^30^, ^31^

Early diagnosis of SLD is crucial, given its growing impact on public health. Timely diagnosis enables interventions to reverse and reduce hepatic steatosis, encompassing lifestyle modifications, structured exercise regime and diet, and bariatric surgery. Effective management of metabolic risk factor such as hypertension, dyslipidaemia and diabetes play a pivotal role in curbing the progression of hepatic and extra‐hepatic complications associated with SLD. Therefore, achieving an accurate and non‐invasive diagnosis of hepatic steatosis is imperative for proactive and comprehensive disease management.

Recent years have witnessed several studies delving into the comparative diagnostic performance of various quantitative ultrasound parameters and subjective ultrasound parameters. While MRI PDFF stands out as the most accurate non‐invasive imaging modality for hepatic steatosis across the disease spectrum, its widespread adoption is curtailed by factors such as cost and limited availability as a point‐of‐care tool.^5^, ^13^ Earlier investigations have pitted CAP against ultrasound in detecting hepatic steatosis with CAP showcasing superior performance, especially in patients with chronic viral hepatitis and advanced liver fibrosis.^32^ It is noteworthy that the general review of CAP has been positive, with guidelines recommending CAP as the first line imaging tool for rapid and standardised hepatic steatosis assessment. However, some have pointed out its limitations, particularly some inadequacy in grading steatosis and the varied disease and probe‐defined cut‐offs.^5^, ^6^, ^33^

To date, this is the only study that compares the correlation between subjective parameters, ATI and histologic steatosis grading. In this study, most of the examined ultrasound parameters demonstrated a robust correlation with the reference standard of histologic diagnosis and grading which is consistent with the existing literature.^13^, ^15^ We found that the radiologist's overall impression had the strongest correlation with histologic steatosis grade, r = 0.82, emphasising the importance of operator experience for steatosis assessment. This differs from an earlier study by Hong et al. that found that LHVB most strongly correlated with histologic grade compared to other subjective ultrasound parameters.^34^ In our study, other qualitative parameters, liver–kidney contrast (r = 0.80, p < 0.001) and Hamaguchi score (r = 0.79, p < 0.001) also displayed very strong correlation with histologic grade which is consistent with other studies where Hamaguchi Score was shown to have an AUC of > 0.98% for detection of NAFLD.^19^ Liver echotexture and focal fat sparing were the only two variables which had poor correlation with histologic steatosis grade. As one might expect, liver echotexture was more closely related to hepatic fibrosis than steatosis.

Attenuation imaging has been shown to have good correlation with histologic steatosis grading in multiple studies. Excellent intraobserver and interobserver reproducibility has also been shown.^23^, ^24^, ^27^, ^35^ One study with 76 adult patients found that ATI had an AUC of 0.85 to detect any steatosis (S1–S3) and an AUC of 0.91 for detecting moderate to severe steatosis (S2–S3).^24^ About 60% of our patients had no to mild steatosis (S0–S1) on liver biopsy and this might account for the reduced correlation of ATI to steatosis grade (r = 0.70, P < 0.001) compared to the other examined subjective US parameters.

Qualitative ultrasound parameters have also been found to excel in detecting moderate to severe hepatic steatosis, though their diagnostic power diminishes in cases of mild steatosis.^15^, ^16^ Unlike quantitative ultrasound parameters, qualitative counterparts exhibit limited clinical utility for assessing severity of steatosis, making them less recommended as a primary imaging approach according to the guidelines for hepatic steatosis assessment.^5^, ^6^ Previous studies including research by Saadeh et al. have demonstrated the inadequacy of qualitative US in distinguishing NASH and nonprogressive NAFLD, highlighting a lack of sensitivity in discriminating the degree of steatosis.^18^ Notably, our study, despite a majority of patients (60%) exhibiting no to mild steatosis, demonstrated a strong correlation between most qualitative parameters and histologic steatosis grade. This correlation may be attributed to the experience of the operator and radiologist involved, emphasising pivotal role of expertise in achieving accurate qualitative ultrasound assessment. Importantly, these findings have underscored the ongoing and valuable role that qualitative ultrasound continues to play in the comprehensive assessment of hepatic steatosis, especially considering its high availability and proficiency in the clinical setting.

One of the reported disadvantages of B‐mode ultrasound has been the high interobserver variability in the interpretation of steatosis due to its high operator dependence.^13^, ^34^, ^36^ B‐mode ultrasound was found to have a varied interobserver agreement of 0.208–0.225 and an intraobserver agreement of 0.356–0.591.^36^ Notably, our study consists of the largest cohort to date in assessing interobserver variability of B‐mode ultrasound in hepatic steatosis assessment. We found that most subjective parameters had moderate to near perfect interobserver agreement with *k* > 0.80. This differs from a more recent study by Hong et al. that assessed similar ultrasound parameters including large hepatic vein blurring, liver–kidney contrast and diaphragm definition. The study showed fair to moderate interobserver agreement with an intraclass correlation coefficient ranging from 0.014 to the highest agreement of only 0.540 with intraobserver agreement ranging from 0.430 to 0.77.^34^ Interestingly, the radiologist's overall impression with three observers' rating had near perfect agreement (Kendall coefficient of 0.90, P < 0.001). From our results, the high interobserver reliability suggests an ongoing role for B‐mode ultrasound in cross‐sectional assessment of hepatic steatosis, though limitations are likely to remain for smaller increments of change in longitudinal assessments in people undertaking lifestyle or pharmacological interventions.

There were a few limitations in our study. Firstly, this was a single centre retrospective study, and our study population may not represent the broader, general population. Most of the patients included were suspected to have severe hepatic disease and were attending a hospital setting. Secondly, there was limited or missing data for some patients, precluding grading of some qualitative variables, especially focal fatty sparing, as well as HRI as most of our patients did not have that data at the time of the biopsy. Thirdly, as a retrospective study, the results might have been affected by other confounding factors that were not adjusted for. For example, interpretation of the ultrasound images and the histologic grade, and therefore the correlation between them may have been influenced by the quality of the captured images and the quality of the biopsy. Lastly, there is potentially variation between the attenuation techniques utilised by different manufacturers, and by differences between models from the same manufacturer, due to incremental technological improvements. Numerical results in this study are Canon and unit of measurement‐specific and may not be applicable to other manufacturers.

Future studies with a larger and more diverse population should focus on further standardising quantitative and qualitative US parameters for hepatic steatosis assessment.

## 5 Conclusion

The examined quantitative and qualitative ultrasound parameters have good to excellent performance for diagnosing clinically significant hepatic steatosis. In the hands of experienced operators, the radiologist's overall impression is at least as good as quantitative parameters for the assessment of hepatic steatosis. This study found that there is an ongoing role of B‐mode ultrasound in the primary diagnosis and grading of steatotic liver disease. Qualitative ultrasound assessment, which is still more commonly used and easier to perform in a clinical setting, along with newer objective measures, will be able to provide a more comprehensive evaluation of hepatic steatosis with better reliability and accuracy.

### 5.1 Authorship statement

The authorship listing conforms with the journal's authorship policy, and that all authors are in agreement with the content of the submitted manuscript.

## 6 Funding

No funding information is provided.

## 7 Conflict of interest

The authors declare no conflict of interest and no financial relationship to disclose.

## Supporting information


**Table S1.** Grading score used for subjective ultrasound parameters (1, 2, 3, 4).
**Table S2.** Grading for Hamaguchi score (5).
**Table S3.** Hepatorenal index cut‐off values for liver steatosis grades (6, 7).
**Table S4.** Controlled attenuation parameter cut‐off values for liver steatosis grades (8).
**Table S5.** Attenuation coefficient cut‐off values according to liver steatosis grade (9, 10).
**Table S6.** Criteria for grading hepatic steatosis based on qualitative B‐mode features (11).

## References

[1] Younossi ZM , Golabi P , Paik JM , Henry A , van Dongen C , Henry L . The global epidemiology of nonalcoholic fatty liver disease (NAFLD) and nonalcoholic steatohepatitis (NASH): a systematic review. Hepatology 2023; 77(4): 1335–1347. 36626630 10.1097/HEP.0000000000000004 PMC10026948

[2] Rinella ME , Lazarus JV , Ratziu V , Francque SM , Sanyal AJ , Kanwal F , *et al*. A multi‐society Delphi consensus statement on new fatty liver disease nomenclature. Hepatology 2023; 78(6): 1966–1986.37363821

[3] Byrne CD , Targher G . NAFLD: a multisystem disease. J Hepatol 2015; 62(1 Suppl): S47–S64. 25920090 10.1016/j.jhep.2014.12.012

[4] McCaughan GW , Munn SR . Liver transplantation in Australia and New Zealand. Liver Transpl 2016; 22(6): 830–838. 27028552 10.1002/lt.24446

[5] Rinella ME , Neuschwander‐Tetri BA , Siddiqui MS , Abdelmalek MF , Caldwell S , Barb D , *et al*. AASLD practice guidance on the clinical assessment and management of nonalcoholic fatty liver disease. Hepatology 2023; 77(5): 1797–1835. 36727674 10.1097/HEP.0000000000000323 PMC10735173

[6] European Association for the Study of the Liver. Electronic address: easloffice@easloffice.eu; Clinical Practice Guideline Panel; Chair; EASL Governing Board representative; Panel members . EASL Clinical Practice Guidelines on non‐invasive tests for evaluation of liver disease severity and prognosis – 2021 update. J Hepatol 2021; 75(3): 659–689. 34166721 10.1016/j.jhep.2021.05.025

[7] Ando Y , Jou JH . Nonalcoholic fatty liver disease and recent guideline updates. Clin Liver Dis (Hoboken) 2021; 17(1): 23–28. 33552482 10.1002/cld.1045 PMC7849298

[8] Kleiner DE , Brunt EM , van Natta M , Behling C , Contos MJ , Cummings OW , *et al*. Design and validation of a histological scoring system for nonalcoholic fatty liver disease. Hepatology 2005; 41(6): 1313–1321. 15915461 10.1002/hep.20701

[9] Takahashi Y , Fukusato T . Histopathology of nonalcoholic fatty liver disease/nonalcoholic steatohepatitis. World J Gastroenterol 2014; 20(42): 15539–15548. 25400438 10.3748/wjg.v20.i42.15539 PMC4229519

[10] Ratziu V , Charlotte F , Heurtier A , Gombert S , Giral P , Bruckert E , *et al*. Sampling variability of liver biopsy in nonalcoholic fatty liver disease. Gastroenterology 2005; 128(7): 1898–1906. 15940625 10.1053/j.gastro.2005.03.084

[11] Adams LA , Roberts SK , Strasser SI , Mahady SE , Powell E , Estes C , *et al*. Nonalcoholic fatty liver disease burden: Australia, 2019–2030. J Gastroenterol Hepatol 2020; 35(9): 1628–1635. 32048317 10.1111/jgh.15009 PMC7540570

[12] Zhou JH , Cai JJ , She ZG , Li HL . Noninvasive evaluation of nonalcoholic fatty liver disease: current evidence and practice. World J Gastroenterol 2019; 25(11): 1307–1326. 30918425 10.3748/wjg.v25.i11.1307 PMC6429343

[13] Ferraioli G , Soares Monteiro LB . Ultrasound‐based techniques for the diagnosis of liver steatosis. World J Gastroenterol 2019; 25(40): 6053–6062. 31686762 10.3748/wjg.v25.i40.6053 PMC6824276

[14] Zeng KY , Bao WYG , Wang YH , Liao M , Yang J , Huang JY , *et al*. Non‐invasive evaluation of liver steatosis with imaging modalities: new techniques and applications. World J Gastroenterol 2023; 29(17): 2534–2550. 37213404 10.3748/wjg.v29.i17.2534 PMC10198053

[15] Hernaez R , Lazo M , Bonekamp S , Kamel I , Brancati FL , Guallar E , *et al*. Diagnostic accuracy and reliability of ultrasonography for the detection of fatty liver: a meta‐analysis. Hepatology 2011; 54(3): 1082–1090. 21618575 10.1002/hep.24452 PMC4197002

[16] Petzold G , Lasser J , Rühl J , Bremer SCB , Knoop RF , Ellenrieder V , *et al*. Diagnostic accuracy of B‐mode ultrasound and hepatorenal index for graduation of hepatic steatosis in patients with chronic liver disease. PLoS One 2020; 15(5): e0231044. 32357147 10.1371/journal.pone.0231044 PMC7194436

[17] Dasarathy S , Dasarathy J , Khiyami A , Joseph R , Lopez R , McCullough AJ . Validity of real time ultrasound in the diagnosis of hepatic steatosis: a prospective study. J Hepatol 2009; 51(6): 1061–1067. 19846234 10.1016/j.jhep.2009.09.001 PMC6136148

[18] Saadeh S , Younossi ZM , Remer EM , Gramlich T , Ong JP , Hurley M , *et al*. The utility of radiological imaging in nonalcoholic fatty liver disease. Gastroenterology 2002; 123(3): 745–750. 12198701 10.1053/gast.2002.35354

[19] Hamaguchi M , Kojima T , Itoh Y , Harano Y , Fujii K , Nakajima T , *et al*. The severity of ultrasonographic findings in nonalcoholic fatty liver disease reflects the metabolic syndrome and visceral fat accumulation. Am J Gastroenterol 2007; 102(12): 2708–2715. 17894848 10.1111/j.1572-0241.2007.01526.x

[20] Webb M , Yeshua H , Zelber‐Sagi S , Santo E , Brazowski E , Halpern Z , *et al*. Diagnostic value of a computerized hepatorenal index for sonographic quantification of liver steatosis. AJR Am J Roentgenol 2009; 192(4): 909–914. 19304694 10.2214/AJR.07.4016

[21] Kozlowska‐Petriczko K , Wunsch E , Petriczko J , Syn WK , Milkiewicz P . Diagnostic accuracy of non‐imaging and ultrasound‐based assessment of hepatic steatosis using controlled attenuation parameter (CAP) as reference. J Clin Med 2021; 10(7): 1507. 33916626 10.3390/jcm10071507 PMC8038574

[22] Tada T , Iijima H , Kobayashi N , Yoshida M , Nishimura T , Kumada T , *et al*. Usefulness of attenuation imaging with an ultrasound scanner for the evaluation of hepatic steatosis. Ultrasound Med Biol 2019; 45(10): 2679–2687. 31277922 10.1016/j.ultrasmedbio.2019.05.033

[23] Jeon SK , Lee JM , Joo I , Yoon JH , Lee DH , Lee JY , *et al*. Prospective evaluation of hepatic steatosis using ultrasound attenuation imaging in patients with chronic liver disease with magnetic resonance imaging proton density fat fraction as the reference standard. Ultrasound Med Biol 2019; 45(6): 1407–1416. 30975533 10.1016/j.ultrasmedbio.2019.02.008

[24] Welman CJ , Saunders J , Zelesco M , Abbott S , Boardman G , Ayonrinde OT . Hepatic steatosis: ultrasound assessment using attenuation imaging (ATI) with liver biopsy correlation. J Med Imaging Radiat Oncol 2023; 67(1): 45–53. 35466506 10.1111/1754-9485.13412

[25] Ferraioli G , Maiocchi L , Raciti MV , Tinelli C , de Silvestri A , Nichetti M , *et al*. Detection of liver steatosis with a novel ultrasound‐based technique: a pilot study using MRI‐derived proton density fat fraction as the gold standard. Clin Transl Gastroenterol 2019; 10(10): e00081. 31609745 10.14309/ctg.0000000000000081 PMC6884349

[26] Huang YL , Bian H , Zhu YL , Yan HM , Wang WP , Xia MF , *et al*. Quantitative diagnosis of nonalcoholic fatty liver disease with ultrasound attenuation imaging in a biopsy‐proven cohort. Acad Radiol 2023; 30(Suppl 1): S155–S163. 37407373 10.1016/j.acra.2023.05.033

[27] D'Hondt A , Rubesova E , Xie H , Shamdasani V , Barth RA . Liver fat quantification by ultrasound in children: a prospective study. AJR Am J Roentgenol 2021; 217(4): 996–1006. 33438457 10.2214/AJR.20.24874

[28] Zhu Y , Yin H , Zhou D , Zhao Q , Wang K , Fan Y , *et al*. A prospective comparison of three ultrasound‐based techniques in quantitative diagnosis of hepatic steatosis in NAFLD. Abdom Radiol (NY) 2024; 49(1): 81–92. 37950767 10.1007/s00261-023-04078-7

[29] Paik JM , Golabi P , Younossi Y , Mishra A , Younossi ZM . Changes in the global burden of chronic liver diseases from 2012 to 2017: the growing impact of NAFLD. Hepatology 2020; 72(5): 1605–1616. 32043613 10.1002/hep.31173

[30] Ayonrinde OT . Historical narrative from fatty liver in the nineteenth century to contemporary NAFLD – reconciling the present with the past. JHEP Rep 2021; 3(3): 100261. 34036255 10.1016/j.jhepr.2021.100261 PMC8135048

[31] Huang DQ , El‐Serag HB , Loomba R . Global epidemiology of NAFLD‐related HCC: trends, predictions, risk factors and prevention. Nat Rev Gastroenterol Hepatol 2021; 18(4): 223–238. 33349658 10.1038/s41575-020-00381-6 PMC8016738

[32] Ferraioli G , Tinelli C , de Silvestri A , Lissandrin R , Above E , Dellafiore C , *et al*. The clinical value of controlled attenuation parameter for the noninvasive assessment of liver steatosis. Liver Int 2016; 36(12): 1860–1866. 27439331 10.1111/liv.13207

[33] Ferraioli G . CAP for the detection of hepatic steatosis in clinical practice. Lancet Gastroenterol Hepatol 2021; 6(3): 151–152. 33460568 10.1016/S2468-1253(20)30367-8

[34] Hong CW , Marsh A , Wolfson T , Paige J , Dekhordy SF , Schlein AN , *et al*. Reader agreement and accuracy of ultrasound features for hepatic steatosis. Abdom Radiol (NY) 2019; 44(1): 54–64. 29951900 10.1007/s00261-018-1683-0 PMC6310678

[35] Yoo J , Lee JM , Joo I , Lee DH , Yoon JH , Kang HJ , *et al*. Reproducibility of ultrasound attenuation imaging for the noninvasive evaluation of hepatic steatosis. Ultrasonography 2020; 39(2): 121–129. 31693842 10.14366/usg.19034 PMC7065988

[36] Cengiz M , Sentürk S , Cetin B , Bayrak AH , Bilek SU . Sonographic assessment of fatty liver: intraobserver and interobserver variability. Int J Clin Exp Med 2014; 7(12): 5453–5460. 25664055 PMC4307502

