# Supplementary material for: Hepatic steatosis: Qualitative and quantitative sonographic assessment in comparison to histology
Source: Australas J Ultrasound Med. 2024 Apr 25;27(3):179–88. doi: 10.1002/ajum.12381 (PMC11423484; doi:10.1002/ajum.12381)
Supplement: Supplementary file 1 — Table S1. Grading score used for subjective ultrasound parameters (1, 2, 3, 4). Table S2. Grading for Hamaguchi score (5). Table S3. Hepatorenal index cut‐off values for liver steatosis grades (6, 7). Table S4. Controlled attenuation parameter cut‐off values for liver steatosis grades (8). Table S5. Attenuation coefficient cut‐off values according to liver steatosis grade (9, 10). Table S6. Criteria for grading hepatic steatosis based on qualitative B‐mode features (11). [file AJUM-27-179-s001.docx]

**Supplementary tables**

Supplementary table 1: Grading score used for subjective Ultrasound parameters (1, 2, 3, 4)

| **Ultrasound B-mode imaging Grading** | **Sonographic features** |
| --- | --- |
| Normal | Normal liver echotexture (slightly more echogenic than right renal) |
| Mild | Diffuse increase in fine echoes in liver parenchyma with normal visualization of diaphragm and intrahepatic vessel borders (OR – significantly more echogenic than renal cortex, but with preserved vascular wall conspicuity) |
| Moderate | Diffuse increase in fine echoes with slightly impaired visualization of intrahepatic vessels and diaphragm (OR – reduced vascular wall conspicuity, but largely preserved through-transmission) |
| Severe (1) | Marked increase in fine echoes with poor or non-visualization of the intrahepatic vessel borders, diaphragm, and posterior right lobe of the liver (OR – significantly reduced through-transmission, not due to other technical factors) |

Supplementary table 2: Grading for Hamaguchi score (5)

| Features | Score | Criteria |
| --- | --- | --- |
| 1. Bright Liver and hepatorenal echo contrast | 0 | Bright liver and hepatorenal echo contrast were negative |
|  | 1 | Either bright liver was positive or hepatorenal echo contrast was positive |
|  | 2 | Bright liver was mild, and hepatorenal echo contrast was positive |
|  | 3 | Bright liver was severe, and hepatorenal echo contrast was positive |
| 1. Deep Attenuation | 0 | Deep Attenuation was negative |
|  | 1 | Visualisation of the diaphragm was obscure, but an observer could distinguish the diaphragm |
|  | 2 | An observer couldn’t distinguish the diaphragm |
| 1. Vessel Blurring | 0 | Vessel blurring was negative |
|  |  | The borders of intrahepatic vessels were unclear and the lumen of intrahepatic vessels was narrowed |
| Sum score of A, B and C if score of A is more than one  Total score is 0, if score of A is 0 | | |

Supplementary table 3: Hepatorenal Index (HRI) cut-off values for liver steatosis grades (6, 7)

| **Liver Steatosis Grade** | **HRI cut-off values** |
| --- | --- |
| 1 | 1.49 |
| 2 | 1.86 |
| 3 | 2.23 |

Supplementary table 4: Controlled Attenuation Parameter (CAP) cut-off values for liver steatosis grades (8)

| **Liver steatosis grade** | **CAP cut off values**  **(dB/m)** |
| --- | --- |
| 1 | 274.8 |
| 2 | 281.0 |
| 3 | 313.1 |

Supplementary table 5: Attenuation Coefficient (ATI) cut-off values according to liver steatosis grade (9, 10)

| **Liver steatosis grade** | **ATI cut off values**  **(dB/cm/MHz)** |
| --- | --- |
| 1 | 0.69 |
| 2 | 0.75 |
| 3 | 0.89 |

**Scoring/Grading of images:**

Supplementary table 6: Criteria for grading hepatic steatosis based on qualitative B-mode features (11)

| Features | Grade | Criteria |
| --- | --- | --- |
| Large Hepatic Vein Blurring | 0: No blurring | Large hepatic veins are clearly visualised and walls are sharply defined |
|  | 1: Mild-moderate blurring | Large hepatic veins are clearly visualised but walls are not sharply defined |
|  | 2: Severe blurring | Large hepatic veins are not clearly visualized |
| Main and Right Portal Vein Blurring | 0: No blurring | Main and right portal veins are clearly visualized, and walls are sharply defined |
|  | 1: Mild-moderate blurring | Main and right portal veins are clearly visualized but walls are not sharply defined |
|  | 2: Severe blurring | Main and right portal veins are not clearly visualized |
| Anterior and Posterior division Right Portal Vein Blurring | 0: No blurring | Anterior and posterior portal veins are clearly visualised and walls are sharply defined |
|  | 1: Mild-moderate blurring | Anterior and posterior portal veins are clearly visualised but walls are not sharply defined |
|  | 2: Severe blurring | Anterior and posterior portal veins are not clearly visualised |
| Liver-Kidney Contrast | 0: Isoechoic | Right liver lobe parenchyma is isoechoic compared to right kidney cortex |
|  | 1: Mildly hyperechoic | Right liver lobe parenchyma is mildly hyperechoic compared to right kidney cortex (requires careful inspection, not dramatically brighter). |
|  | 2: Moderately hyperechoic | Right liver lobe parenchyma is moderately hyperechoic compared to right kidney cortex (immediately apparent, but not dramatically brighter) |
|  | 3: Markedly hyperechoic | Right liver lobe parenchyma is markedly hyperechoic compared to right kidney cortex (immediately apparent and dramatically brighter) |
|  | Not applicable | Right kidney not visualized or visible but clearly abnormal |
| Posterior Beam Attenuation | 0: No attenuation | No definite posterior beam attenuation |
|  | 1: Mild-moderate attenuation | Definite posterior beam attenuation but not dramatic |
|  | 2: Markedly hyperechoic | Definite and dramatic posterior beam attenuation |
| Diaphragm Definition | 0: Clearly defined | Diaphragm visualised in its entirety as a sharp line |
|  | 1: Obscured | Diaphragm visualised as an interrupted or blurry line |
|  | 2: Obliterated | Diaphragm not visualised at all |
| Focal Fat Sparing | 0: Absent | Focal fat sparing is absent |
|  | 1: Present | Focal geographic hypoechoic area(s) observed adjacent to gallbladder wall or portal vessel wall |
| Liver Echotexture | 0: Normal | Normal |
|  | 1: Abnormal | Coarse echoes |
| Overall Impression | 0: No hepatic steatosis | Based on subjective interpretation |
|  | 1: Mild hepatic steatosis | Based on subjective interpretation |
|  | 2: Moderate hepatic steatosis | Based on subjective interpretation |
|  | 3: Severe hepatic steatosis | Based on subjective interpretation |

Additional References:

1. Ferraioli G, Soares Monteiro LB. Ultrasound-based techniques for the diagnosis of liver steatosis. World J Gastroenterol. 2019;25(40):6053-62.

2. Hernaez R, Lazo M, Bonekamp S, Kamel I, Brancati FL, Guallar E, et al. Diagnostic accuracy and reliability of ultrasonography for the detection of fatty liver: a meta-analysis. Hepatology. 2011;54(3):1082-90.

3. Dasarathy S, Dasarathy J, Khiyami A, Joseph R, Lopez R, McCullough AJ. Validity of real time ultrasound in the diagnosis of hepatic steatosis: a prospective study. J Hepatol. 2009;51(6):1061-7.

4. Saadeh S, Younossi ZM, Remer EM, Gramlich T, Ong JP, Hurley M, et al. The utility of radiological imaging in nonalcoholic fatty liver disease. Gastroenterology. 2002;123(3):745-50.

5. Hamaguchi M, Kojima T, Itoh Y, Harano Y, Fujii K, Nakajima T, et al. The severity of ultrasonographic findings in nonalcoholic fatty liver disease reflects the metabolic syndrome and visceral fat accumulation. Am J Gastroenterol. 2007;102(12):2708-15.

6. Webb M, Yeshua H, Zelber-Sagi S, Santo E, Brazowski E, Halpern Z, et al. Diagnostic value of a computerized hepatorenal index for sonographic quantification of liver steatosis. AJR Am J Roentgenol. 2009;192(4):909-14.

7. Xia MF, Yan HM, He WY, Li XM, Li CL, Yao XZ, et al. Standardized ultrasound hepatic/renal ratio and hepatic attenuation rate to quantify liver fat content: an improvement method. Obesity (Silver Spring). 2012;20(2):444-52.

8. Cao YT, Xiang LL, Qi F, Zhang YJ, Chen Y, Zhou XQ. Accuracy of controlled attenuation parameter (CAP) and liver stiffness measurement (LSM) for assessing steatosis and fibrosis in non-alcoholic fatty liver disease: A systematic review and meta-analysis. EClinicalMedicine. 2022;51:101547.

9. Tada T, Iijima H, Kobayashi N, Yoshida M, Nishimura T, Kumada T, et al. Usefulness of Attenuation Imaging with an Ultrasound Scanner for the Evaluation of Hepatic Steatosis. Ultrasound Med Biol. 2019;45(10):2679-87.

10. Jeon SK, Lee JM, Joo I, Yoon JH, Lee DH, Lee JY, et al. Prospective Evaluation of Hepatic Steatosis Using Ultrasound Attenuation Imaging in Patients with Chronic Liver Disease with Magnetic Resonance Imaging Proton Density Fat Fraction as the Reference Standard. Ultrasound Med Biol. 2019;45(6):1407-16.

11. Hong CW, Marsh A, Wolfson T, Paige J, Dekhordy SF, Schlein AN, et al. Reader agreement and accuracy of ultrasound features for hepatic steatosis. Abdom Radiol (NY). 2019;44(1):54-64.
